# Deterioration from healthy to mild cognitive impairment and Alzheimer’s disease mirrored in corresponding loss of centrality in directed brain networks

**DOI:** 10.1186/s40708-019-0101-x

**Published:** 2019-12-02

**Authors:** Sinan Zhao, D. Rangaprakash, Peipeng Liang, Gopikrishna Deshpande

**Affiliations:** 1 AU MRI Research Center, Department of Electrical and Computer Engineering, Auburn University, 560 Devall Dr, Suite 266D, Auburn, AL 36849 USA; 2 Department of Radiology, Northwestern University, Chicago, IL USA; 3 School of Psychology, Capital Normal University, Beijing, China; 4 Department of Psychology, Auburn University, Auburn, AL USA; 5 Alabama Advanced Imaging Consortium, Auburn, AL USA; 6 Center for Neuroscience, Auburn University, Auburn, AL USA; 7 Center for Health Ecology and Equity Research, Auburn University, Auburn, AL USA; 8 Department of Psychiatry, National Institute of Mental Health and Neurosciences, Bangalore, India

**Keywords:** Alzheimer’s disease, Functional MRI, Brain connectivity, Granger causality, Graph theory, Betweenness centrality, Middleman power

## Abstract

**Objective:**

It is important to identify brain-based biomarkers that progressively deteriorate from healthy to mild cognitive impairment (MCI) to Alzheimer’s disease (AD). Cortical thickness, amyloid-ß deposition, and graph measures derived from functional connectivity (FC) networks obtained using functional MRI (fMRI) have been previously identified as potential biomarkers. Specifically, in the latter case, betweenness centrality (BC), a nodal graph measure quantifying information flow, is reduced in both AD and MCI. However, all such reports have utilized BC calculated from undirected networks that characterize synchronization rather than information flow, which is better characterized using directed networks.

**Methods:**

Therefore, we estimated BC from directed networks using Granger causality (GC) on resting-state fMRI data (*N* = 132) to compare the following populations (*p* < 0.05, FDR corrected for multiple comparisons): normal control (NC), early MCI (EMCI), late MCI (LMCI) and AD. We used an additional metric called middleman power (MP), which not only characterizes nodal information flow as in BC, but also measures nodal power critical for information flow in the entire network.

**Results:**

MP detected more brain regions than BC that progressively deteriorated from NC to EMCI to LMCI to AD, as well as exhibited significant associations with behavioral measures. Additionally, graph measures obtained from conventional FC networks could not identify a single node, underscoring the relevance of GC.

**Conclusion:**

Our findings demonstrate the superiority of MP over BC as well as GC over FC in our case. MP obtained from GC networks could serve as a potential biomarker for progressive deterioration of MCI and AD.

## Introduction

Alzheimer’s disease (AD) is a neurodegenerative disorder [1–3] that is initially characterized by memory loss, and then cognitive decline and incapacitation as the disease progresses. Mild cognitive impairment (MCI) presents as a transition period between normal aging and AD, whose characteristics are similar to AD [4]. Approximately 50% of MCI patients transition to AD in 3–5 years [5]. Therefore, to understand disease progression [6, 7], this study is aimed at identifying brain-based biomarkers that progressively deteriorate from healthy to MCI to AD, which will help in diagnosis and interventional treatment.

Resting-state functional magnetic resonance imaging (RS-fMRI) is a promising modality that can non-invasively characterize distributed brain networks [8, 9]. RS-fMRI has been widely used to study the inter-regional functional connectivity (FC) between healthy and disease populations, including for detecting connectivity abnormalities in AD and MCI [10]. Studies have found that AD is associated with alteration of FC among different brain regions [11, 12]. Specifically, it has been shown that AD patients have decreased hippocampal FC with prefrontal lobe and posterior cingulate cortex [13, 14]. Further, connectivity alterations in AD patients’ brain have been shown to occur in medial frontal, medial parietal and posterior cingulate cortex; those regions also exhibit high resting-state metabolism and are part of the “default-mode network” [15]. Huang et al. [16] found that compared with control subjects, AD patients had decreases in the amount of inter-regional FC, especially in the hippocampus, weaker between-lobe FC and between-hemisphere FC. Reduced resting-state FC [17] has been found in the default-mode network of MCI patients. Overall, reduction in connectivity has been reported in MCI and AD. A small number of previous studies have also found increased FC in MCI/AD, which were attributed as compensatory mechanisms for losses in cognitive functionality [18, 19]. There has not been direct and expansive evidence for this alternative model. The deterioration hypothesis (reduced connectivity) is a more mainstream view with wider acceptability since it has roots in molecular/cellular level events in AD [20]; hence, we adopted it in this study.

Connectivity measures are bivariate and ignore how the ensemble of connections characterize brain function, while graph measures quantify the topography of the network, which has been shown to be sensitive to disease processes [21]. Recently, the combination of RS-fMRI and graph theoretical analysis has revealed the topological organization of human whole-brain functional networks. For example, the healthy brain has been shown to exhibit small-world characteristics [21, 22]. Using graph theoretical analysis of AD/MCI patients and healthy populations can lead to better understanding of the differences in the topology of brain networks as well as the relationship between brain connectivity and the disease processes [11, 23]. Previous studies have found widespread reduction in node degree (a measure of connection density) in MCI compared to healthy controls, suggesting that graph-based analyses might potentially be used in the determination of biomarkers for pathological aging [24, 25]. For example, decrease in local clustering coefficient (specifically, in the hippocampus) and increased characteristic path length (CPL) in AD compared to normal controls has been demonstrated [11, 24]. Decreased long-distance connectivity of the frontal and caudal brain regions has been found in AD compared to controls [26]. Moreover, betweenness centrality (BC), a local nodal graph measure that quantifies how much information may traverse the node (any given brain region), was shown to be lower in certain brain regions in both AD and MCI compared to healthy controls [27].

We identify two shortcomings in previous MCI and AD studies employing graph-theoretic complex network analysis of resting-state brain networks. First, it is noteworthy that previous studies have not investigated whether graph measures mirror neuropathological deterioration from NC to MCI to AD. This is important because such metrics could signal a neurodegenerative course, which is different from normal aging at early stages of the disease when intervention is more likely to be successful. Second, previous reports have found promise in BC [27], but have utilized BC calculated from undirected networks that characterize synchronization rather than information flow, which is better characterized using directed networks. It is to be noted that both synchronization and information flow are prevalent yet distinct mechanisms by which brain regions interact with each other. Besides, even though BC can determine the importance of a particular node in a network, it tends to over-inflate the power of nodes [28] as will be explained in the next section.

In this study, we addressed these gaps by estimating BC from directed networks derived from the application of Granger causality (GC) [29–36] to RS-fMRI data acquired from the following populations: Normal Control (NC), Early MCI, Late MCI and AD. We used an additional metric called middleman power (MP) which not only characterizes information flow through a node as in BC, but also estimates the power of the node in terms of its criticality for information flow in the entire network [28]. We hypothesized that BC and MP of some brain regions will progressively decrease [27] from NC to EMCI to LMCI to AD.

## Methods

### Subjects

Data used in this study were obtained from the Alzheimer’s disease neuroimaging initiative (ADNI) database (http://www.loni.ucla.edu/ADNI). ADNI is a multisite, longitudinal observational study of clinical, imaging, genetic and bio-specimen biomarkers through healthy elders to MCI to dementia or AD. The primary goal of ADNI is to assess whether neuroimaging and other markers could be utilized to measure the progression of MCI and AD. Over 800 adults, aged 55–90 years, were recruited from over 50 sites across USA and Canada to be followed for 2 or 3 years.

In this study, RS-fMRI data from 35 control subjects, 34 EMCI, 34 LMCI and 29 AD patients were used from the ADNI-2 section of the database. The participants in this study were recruited between 2011 and 2013 through the ADNI-2 protocol, and we selected subjects who had completed both the 3D MPRAGE and RS-fMRI data scans in the same visit. We manually discarded 2 LMCI patients from the group so that the ages of four groups were statistically matched. Subjects were tested with Neuropsychiatric Inventory Questionnaire (NPI-Q), Mini-mental State Examination (MMSE), Functional Assessment Questionnaire (FAQ), as well as Global Clinical Dementia Rating (Global CDR) (Table 1).

**Table 1:** Demographics and clinical variables

	Controls	EMCI	LMCI	AD
Sex (F/M)	20/15	16/18	14/20	16/13
Age	74.5 ± 5.8	72.2 ± 5.7	71.4 ± 8.6	73.1 ± 7.35
NPI-Q	0.6 ± 1.3	2.1 ± 3.1	2.8 ± 2.5	3.0 ± 2.4
MMSE	28.8 ± 1.6	28.1 ± 1.5	27.1 ± 2.3	20.9 ± 3.9
FAQ	0.2 ± 0.8	3.3 ± 4.1	5.4 ± 6.2	16.3 ± 7.6
Global CDR	0.0 ± 0.1	0.5 ± 0.1	0.5 ± 0.1	0.8 ± 0.2

Functional MRI data were acquired using a T_2_^*^-weighted single shot echo-planar imaging (EPI) sequence on 3.0 Tesla Philips MR scanners with 48 slices, slice thickness = 3.3 mm, TR = 3000 ms, TE = 30 ms, flip angle = 80°, field of view: RL = 212, AP = 198.75 mm, FH = 159 mm, voxel size: RL = 3.3125 mm, AP = 3.3125 mm and 140 temporal volumes in each run. Anatomical images were acquired using magnetization-prepared rapid gradient echo (MPRAGE) sequence for overlay and localization (TR = 6.8 ms, TE = 3.1 ms, voxel size: 1.11 × 1.11 × 1.2 mm^3^, flip angle = 9°, field of view: RL = 204 mm, AP = 253 mm, FH = 270 mm). The data were subjected to a standard resting-state preprocessing pipeline using the Data Processing Assistant for Resting-State fMRI (DPARSF) toolbox that is based on Statistical Parametric Mapping (SPM8) [37, 38]. Mean time series were extracted from 200 functionally homogeneous regions-of-interest (ROIs) identified via spectral clustering (Craddock-200 atlas) [39, 40]. Since our study used functional MRI data, we used this popular functional atlas instead of an anatomical atlas.

### Connectivity analysis

Directional brain networks were obtained from RS-fMRI data using GC [10], [41–46]. The principle underlying GC [47–50] is as follows: If using the past of time series *X* improves the prediction of the future of time series *Y*, then *X* can be said to have a causal influence on *Y* [51]. Let *X*(*t*) = [*x*_1_(*t*), *x*_2_(*t*),…, *x*_*q*_(*t*)] be the *q* selected ROI time series, then the multivariate vector autoregressive (MVAR) model with order *p* is given by

1 $$ X\left( t \right) = \mathop \sum \limits_{n = 1}^{p} A\left( n \right)X\left( {t - n} \right) + E\left( t \right), $$

where *A*(*n*) is the model parameter, and *E*(*t*) is the vector of the residual error. There are many previous studies which have used the MVAR model to estimate the causal relationship between fMRI time series from different brain regions. However, using GC on raw fMRI signals can be confounded by the spatial and inter-subject variability of the hemodynamic response function (HRF) [52–54]. HRF variability is also found to confound group differences in connectivity [55–58], which is of consequence to our study as well. This variability of the HRF and its smoothing effect can be minimized by blind hemodynamic deconvolution methods. Consequently, a popular data-driven blind deconvolution approach based on the detection of pseudo-events proposed by Wu et al. [59] was used to estimate the HRF and latent neuronal time series from the observed data. Specifically, RS-fMRI data were considered as spontaneous and event-related, wherein the events were detected by picking up the comparatively large amplitude of BOLD signal fluctuations after removing other sources of noise. The HRF of each voxel was reconstructed by fitting them with a double gamma function and two time derivatives. Finally, latent neuronal time series were recovered by Wiener deconvolution using the corresponding HRF. When the latent neuronal variables were input into the MVAR model (1) instead of raw fMRI data, we obtained the following equation.

2 $$\begin{aligned} \left[ {\begin{array}{*{20}l} {h_{1} \left( t \right)} \\ {h_{2} \left( t \right)} \\ {\begin{array}{*{20}l} \vdots \\ {h_{q} \left( t \right)} \\ \end{array} } \\ \end{array} } \right] &\quad = \left[ {\begin{array}{*{20}l} {\begin{array}{*{20}l} 0 &\quad {a_{12} \left( 0 \right)} \\ {a_{21} \left( 0 \right)} &\quad 0 \\ \end{array} } &\quad {\begin{array}{*{20}l} \ldots &\quad {a_{1q} \left( 0 \right)} \\ \ldots &\quad {a_{2q} \left( 0 \right)} \\ \end{array} } \\ {\begin{array}{*{20}l} \vdots &\quad \vdots \\ {a_{q1} \left( 0 \right)} &\quad {a_{q2} \left( 0 \right)} \\ \end{array} } &\quad {\begin{array}{*{20}l} 0 &\quad {       \vdots       } \\ \ldots &\quad 0 \\ \end{array} } \\ \end{array} } \right] \times \left[ {\begin{array}{*{20}l} {h_{1} \left( t \right)} \\ {h_{2} \left( t \right)} \\ {\begin{array}{*{20}l} \vdots \\ {h_{q} \left( t \right)} \\ \end{array} } \\ \end{array} } \right] \\ &\quad \quad + \mathop \sum \limits_{n = 1}^{p} \left[ {\begin{array}{*{20}l} {\begin{array}{*{20}c} {a_{11} \left( n \right)} &\quad {a_{12} \left( n \right)} \\ {a_{21} \left( n \right)} &\quad {a_{22} \left( n \right)} \\ \end{array} } &\quad {\begin{array}{*{20}l} \ldots &\quad {a_{1q} \left( n \right)} \\ \ldots &\quad {a_{2q} \left( n \right)} \\ \end{array} } \\ {\begin{array}{*{20}l} \vdots &\quad \vdots \\ {a_{q1} \left( n \right)} &\quad {a_{q2} \left( n \right)} \\ \end{array} } &\quad {\begin{array}{*{20}l} \ddots &\quad \vdots \\ \ldots &\quad {a_{qq} \left( n \right)} \\ \end{array} } \\ \end{array} } \right] \times \left[ {\begin{array}{*{20}l} {h_{1} \left( {t - n} \right)} \\ {h_{2} \left( {t - n} \right)} \\ {\begin{array}{*{20}l} \vdots \\ {h_{q} \left( {t - n} \right)} \\ \end{array} } \\ \end{array} } \right] + \left[ {\begin{array}{*{20}l} {e_{1} \left( t \right)} \\ {e_{2} \left( t \right)} \\ {\begin{array}{*{20}l} \vdots \\ {e_{q} \left( t \right)} \\ \end{array} } \\ \end{array} } \right], \\ \end{aligned} $$

where *h*_*q*_(*t*) are the hidden neural states, *p* is the model order estimated from the Akaike/Bayesian information criterion [10, 31], *a* and *e* are the MVAR model coefficients and errors, respectively. The instantaneous influences between time series are represented by *a*(0) and the causal influences between time series can be inferred from *a*(*n*), *n *= *1*.. *q*. Using *a*(0) in the model can minimize the “leakage” of instantaneous correlation into causality [50, 53, 60, 61]. Subsequently, this correlation-purged Granger causality (CPGC) from time series *j* to time series *i* could be obtained using the following equation

3 $$ {\text{CPGC}}_{ij} = \mathop \sum \limits_{n = 1}^{p} \left\{ {a_{ij} \left( n \right)} \right\}^{2} . $$

The latent neuronal time series corresponding to all the 200 ROIs were first estimated using deconvolution and then input into a first-order MVAR model to obtain the causal connectivity between all pairs of 200 ROI time series. We used a first-order model because causal relationships within neural delays of less than or equal to one TR are of interest in neuroimaging [41]. Since fMRI has a relatively low temporal resolution, a first-order model captures the most relevant causal connectivity information [47]. Surrogate data were obtained by randomizing the phase of the original time series and retaining their magnitude spectrum and then input into the MVAR model. This procedure was repeated 1000 times and the statistical significance of each connection was obtained by comparing the CPGC value obtained from original data with the null distribution obtained from surrogate data. If region A significantly influenced region B (*p* < 0.05), then the path from A to B was considered directionally connected. This way, we obtained the binary directed connectivity matrix for each subject by thresholding the connectivity values (*p* < 0.05, FDR corrected for multiple comparisons). This was used in further graph analysis.

### Nodal graph measures

Betweenness centrality (BC) is a local nodal graph measure that quantifies how much information may traverse the node (any given brain region) and has been widely used in graph analysis [62]. To define the BC measure, let *λ*_*i*_(*st*) be the number of paths [63] between node *s* and node *t*, passing through node *i*. Let the total number of shortest paths between node *s* and node *t* be denoted by *λ*(*st*). Then, the BC in network *D* (containing nodes *s*, *t* and *i*) can be defined as

4 $$ {\text{BC}}_{i} \left( D \right) = \mathop \sum \limits_{s,t: s \ne i \ne t} \frac{{\lambda_{i} \left( {st} \right)}}{{\lambda \left( {st} \right)}}. $$

Equation (4) indicates that nodes with high BC connect, otherwise unconnected parts of the network. However, some nodes located on the shortest path between long distance vertices can turn out to possess rather high values of BC (due to long geodesics) that in actuality are not critical for information flow. This indicates that BC is a rather good local graph measure, but may lose its advantages in large-scale networks. Comparatively speaking, a middleman in a network occupies a critical position that can block at least one node’s information flow to another. In the extreme case, middleman nodes might have the ability to separate the whole network into several disconnected components [28]. On the contrary, centrality measures do not necessarily identify these important critical nodes, even though their removal might cause the functional deterioration of the whole network.

**Fig. 1:**
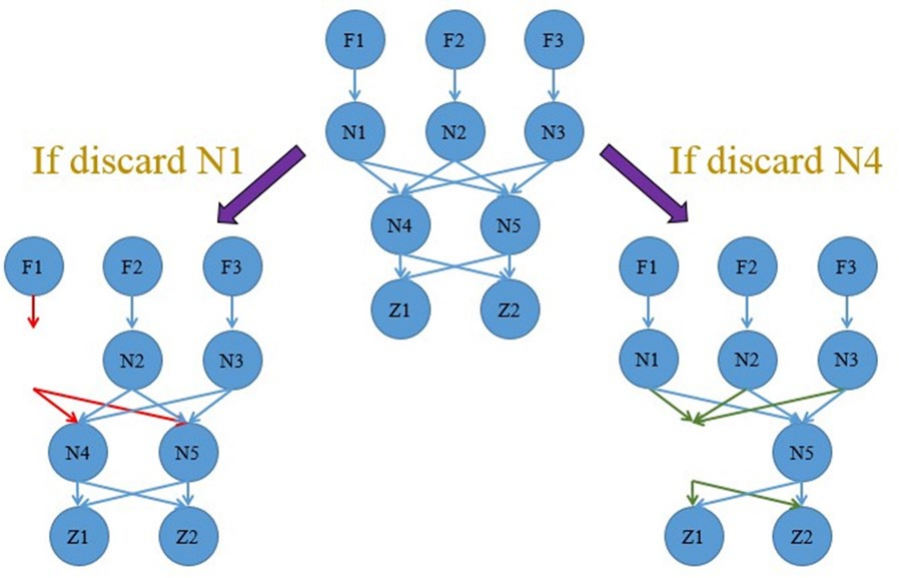
An example network illustrating the concept of middleman power (MP) and its superiority over betweenness centrality (BC) under certain circumstances

For example, consider the directed network shown in Fig. 1. Nodes N1, N2, N3 block information flow from nodes F1, F2, F3 to other nodes, respectively. If we discard node N1 (Fig. 1, left), information flow from F1 to other nodes will be blocked. Thus, the middleman nodes are N1, N2, and N3. The value of un-normalized BC of N1, N2 and N3 is equal to 4. However, if we discard N4 (or N5) separately, it does not block the information flow from any of two nodes that originally communicated with each other (Fig. 1, right). For non-middleman nodes N4 and N5, their BC value is equal to 6. Here, the non-middleman nodes (N4, N5) have higher BC value compared to actual middleman nodes (N1, N2, N3) because betweenness is counted on geodesics, and the geodesics between given nodes have equal weight. This example illustrates that BC tends to exaggerate the power of some non-middleman nodes and thus may not necessarily accurately measure the ‘power’ of middleman nodes (i.e., nodal power), while still measuring nodal information flow (Table 2).

**Table 2:** The value of un-normalized betweenness centrality and middleman power for the directed network in Fig. 1

	Betweenness centrality	Middleman power
N1	4	4
N2	4	4
N3	4	4
N4	6	0
N5	6	0

The brokerage position of middlemen in directed networks allows them to be highly extractive to both directly and indirectly connected nodes. The brokerage of node *i* in network *D* can be defined as

5 $$ b_{i} \left( D \right) = \mathop \sum \limits_{j \in N} \# S_{j} \left( D \right) - \mathop \sum \limits_{j \ne i} \# S_{j} \left( {D - i} \right) - \# S_{i} \left( D \right) - \# P_{i} \left( D \right), $$

where *N* is the set of all nodes in *D*, *i* denotes an arbitrary node belonging to set *N*. Node *j* is the successor of node *i* and node *i* is called the predecessor of *j* if there is at least one node that is adjacent to the path from *i* to *j*. Symbol # denotes the number of instances which satisfy the expression being referred to. For example, #*S*_*j*_(*D*) is the number of successors of node *j* in network D. The maximal potential brokerage in network *D* is defined as

6 $$ B^{\prime}\left( D \right) = \mathop \sum \limits_{i \in N} \left[ {\# S_{i} \left( D \right) - \# s_{i} \left( D \right)} \right], $$

where *s*_*i*_(*D*) are all of the direct successors of *i* in *D*. By normalizing a node’s brokerage score, the middleman power (MP) of a node can be defined as

7 $$ v_{i} \left( D \right) = \frac{{b_{i} \left( D \right)}}{{\hbox{max} \left\{ {B^{\prime}\left( D \right),1} \right\}}}. $$

Equation (7) indicates that if a middleman node breaks all potential opportunity in the network, in other words disconnects connections between all the other nodes due to its removal (such as the removal of the center node of a star shaped network), then the middleman node has a network power of 1. This illustrates that MP measures both nodal information flow and nodal power. In this study, BC [62] and MP [28] graph measures were calculated for each node from the binarized connectivity matrices, obtaining a 200 × 1 BC (and MP) vector per subject.

### Statistical analysis

Six one-sided *t*-tests using BC (and MP) measures were performed (NC > EMCI, NC > LMCI, NC > AD, EMCI > LMCI, EMCI > AD, LMCI > AD), to find common nodes among all the six comparisons to identify brain regions in which BC and MP decreased progressively from NC to EMCI to LMCI and AD (*p* < 0.05, FDR corrected for multiple comparisons, controlled for age and gender).

Besides, as a supplemental analysis, we computed and tested BC for undirected networks (MP is not defined for undirected networks). Conventional FC was computed between each pair of 200 ROI time series using Pearson’s correlation coefficient. The FC matrices were binarized similarly (*p* < 0.05, FDR corrected for multiple comparisons), and a 200 × 1 BC vector was calculated for each subject. Six one-sided t-tests were also performed to find the common nodes as described before (*p* < 0.05, FDR corrected for multiple comparisons).

### Behavioral relevance of nodal graph measures

To determine the behavioral relevance of nodal graph measures, we correlated both MP and BC of ROIs (which satisfied our hypotheses as stated above) with clinical variables (scores of NPI-Q, MMSE, FAQ and Global CDR) using the entire subject sample.

## Results

**Fig. 2:**
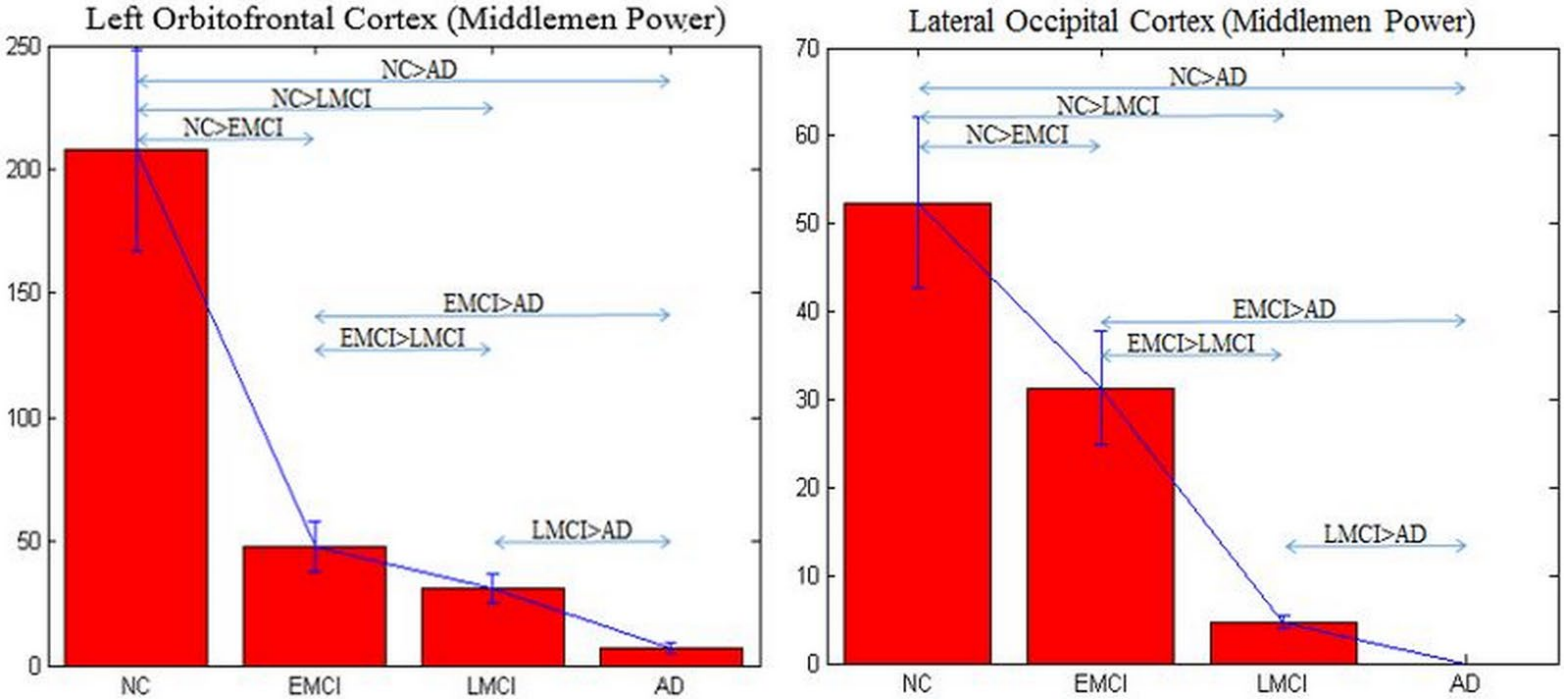
Middleman power (MP) of the left orbitofrontal cortex (L OFC) and lateral occipital cortex (LOC), which were significantly different between the groups and deteriorated from NC to EMCI to LMCI to AD

**Fig. 3:**
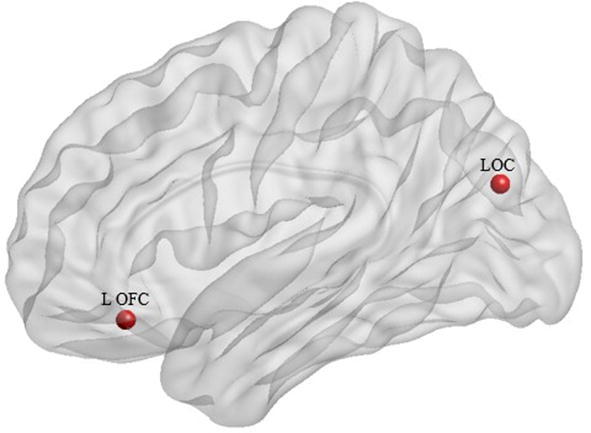
The location of two ROIs in the brain which progressively decreased with the deterioration of disease. L OFC: left orbitofrontal cortex, LOC: lateral occipital cortex

**Fig. 4:**
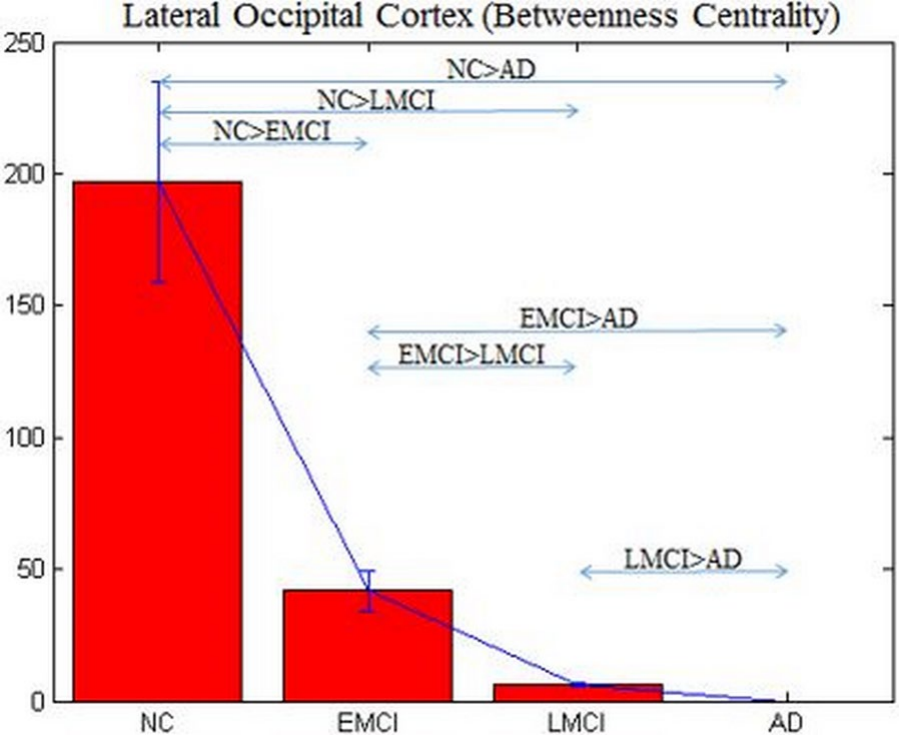
Betweenness centrality (BC) of lateral occipital cortex (LOC) obtained from directed networks using Granger causality (GC), which was significantly different between the groups and deteriorated from NC to EMCI to LMCI to AD

MP of left orbitofrontal cortex (L OFC) and lateral occipital cortex (LOC) progressively decreased from NC to EMCI to LMCI to AD (Fig. 2). These two regions are displayed (Fig. 3) on a brain surface using the BrainNet Viewer visualization tool (http://www.nitrc.org/projects/bnv/) [64]. BC was able to identify only the LOC and not L OFC (Fig. 4).

**Fig. 5:**
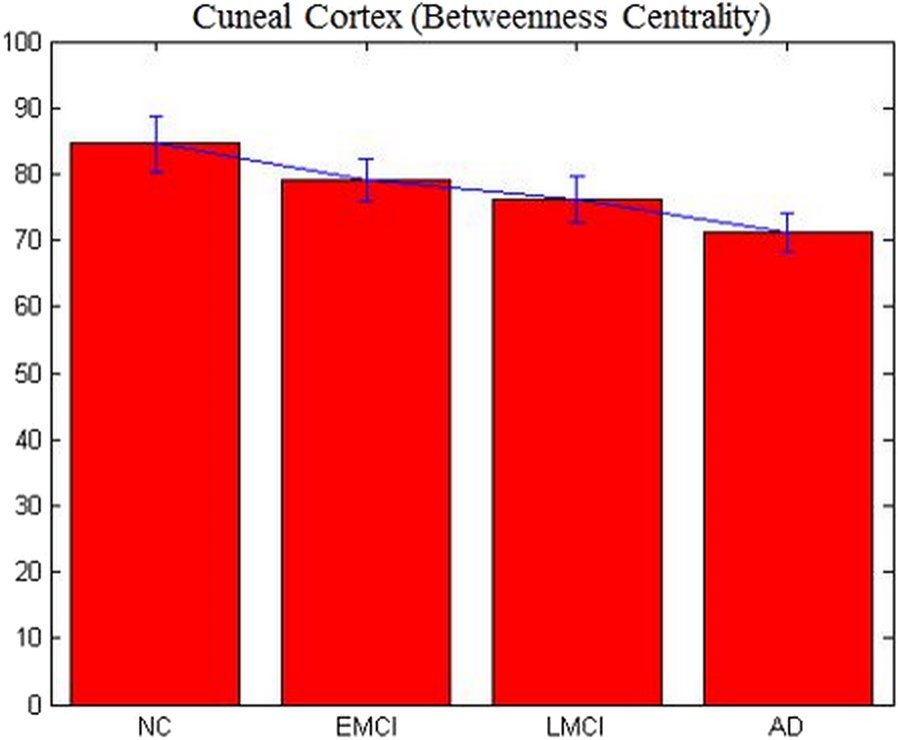
Betweenness centrality (BC) of cuneus obtained from undirected networks using functional connectivity (Pearson’s correlation), which was not significantly different between the groups (*p* > 0.25)

**Fig. 6:**
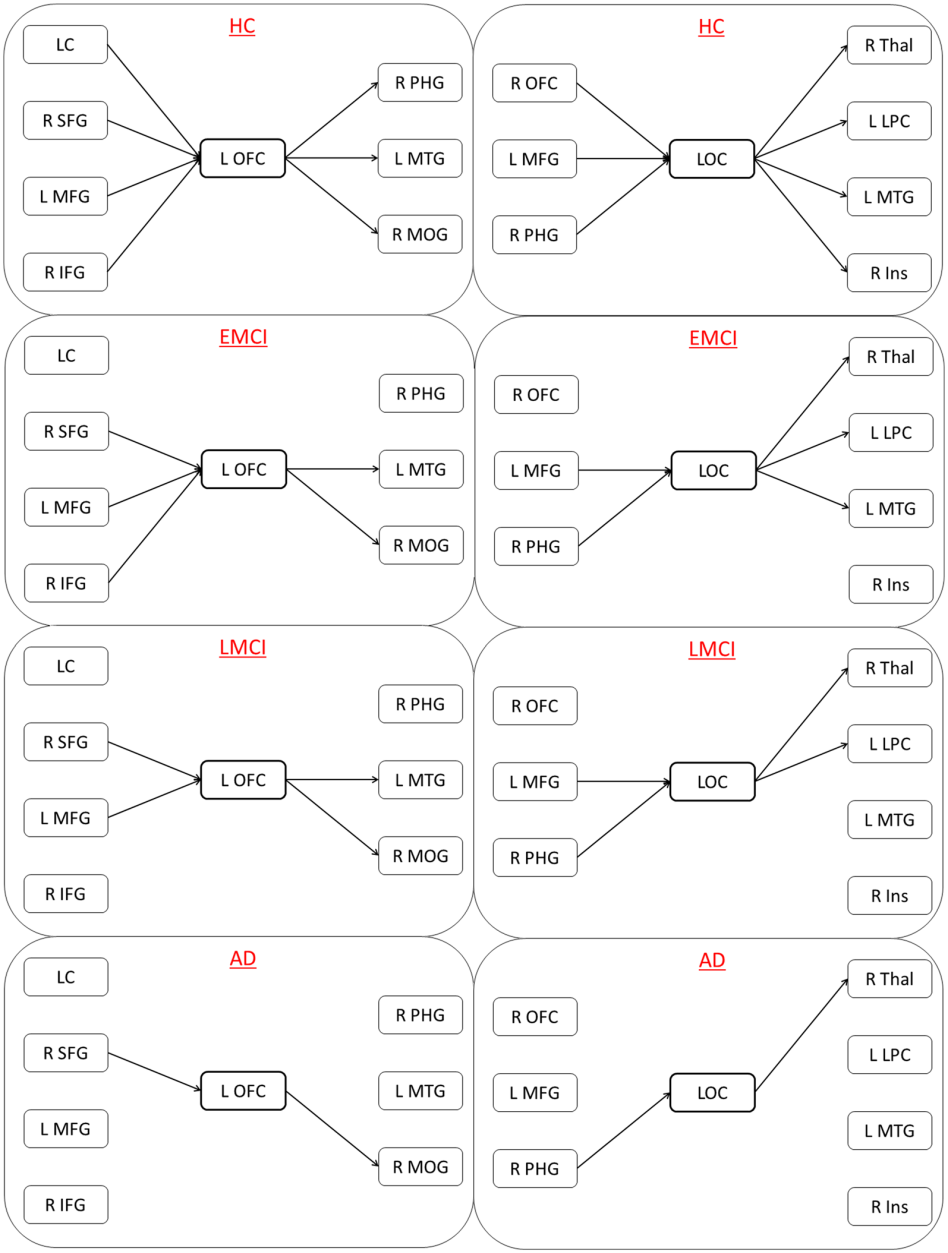
Middleman nodes L OFC (left) and LOC (right) with corresponding incoming and outgoing connections. From top to bottom: HC, EMCI, LMCI, AD. We can observe gradual pruning of the connections associated with these middleman nodes as the disease progresses from EMCI through AD. *OFC* orbito-frontal cortex, *LOC* lateral occipital cortex, *LC* locus coeruleus, *SFG* superior frontal gyrus, *MFG* middle frontal gyrus, *IFG* inferior frontal gyrus, *PHG* parahippocampal gyrus, *MTG* middle temporal gyrus, *MOG* middle occipital gyrus, *Thal* thalamus, *LPS* lateral parietal cortex, *Ins* insula

BC estimated from undirected networks obtained using conventional FC did not identify a single node. We then gradually relaxed the *p* value threshold in all tests and tried to find the node that was common among all the six comparisons. It was not until the p-value threshold was 0.25, that the first node, cuneus (Fig. 5) was identified. Obviously, it was not statistically significant. This demonstrates the superiority of using directed connectivity networks (GC) over conventional FC, as well as the importance of MP over BC obtained from both directed and undirected networks. Figure 6 shows the L OFC and LOC as middleman nodes with incoming and outgoing healthy connections in the HC group, and subsequent gradual pruning of these connections (and the middleman property of these nodes) in EMCI, LMCI and AD groups.

MP of L OFC and LOC, and BC of LOC (all of which were estimated from directed networks), which progressively decreased from NC to EMCI to LMCI to AD, were significantly associated with behavioral measures across the entire subject sample, thus highlighting their relevance to the underlying neuropathology (Tables 3, 4). It is noteworthy that MMSE was highest in controls and lowest in AD; while the opposite was the case for the other three measures. Therefore, it makes sense that MMSE was positively correlated with nodal graph measures, while the other three behavioral measures were negatively correlated. The correlations with behavior were also stronger for MP as compared to BC.

**Table 3:** Correlation value (*R*) and corresponding *p* value for the association between behavioral measures and middleman power of L OFC and LOC

Behavioral measures	L orbitofrontal cortex		Lateral occipital cortex	
	*R*	*p* value	*R*	*p* value
NPI-Q	− 0.34	6.32 × 10^−04^	− 0.38	1.49 × 10^−04^
MMSE	0.41	3.30 × 10^−05^	0.38	1.41 × 10^−04^
FAQ	− 0.48	8.97 × 10^−07^	− 0.46	2.45 × 10^−06^
Global CDR	− 0.79	2.16 × 10^−21^	− 0.83	1.33 × 10^−25^

**Table 4:** Correlation value (*R*) and corresponding *p*-value for the association between behavioral measures and betweenness centrality of LOC

Behavioral measures	Lateral occipital cortex	
	*R*	*p* value
NPI-Q	− 0.24	1.86×10^−02^
MMSE	0.34	1.52×10^−03^
FAQ	− 0.36	3.81×10^−04^
Global CDR	− 0.45	3.11×10^−06^

Finally, as an exploratory analysis, we performed machine learning classification using a linear support vector machine (SVM) classifier [65, 66] to assess the predictive ability of BC and MP measures. Classifying AD vs NC, we found that BC resulted in an accuracy of 92.08%, while MP resulted in 84.97% accuracy. Likewise, classifying EMCI vs NC, BC resulted in 91.79% accuracy while MP resulted in 92.08% accuracy; classifying LMCI vs NC, BC resulted in 92.84% accuracy while MP resulted in 99.01% accuracy. For each comparison, the accuracies of BC and MP were significantly different (*p* < 0.05). It must be noted that, given the modest sample size we had, it would be better to interpret relative differences in classification accuracies rather than their absolute values. It is expected that with larger samples, the absolute values of accuracies may be lesser [67].

The above results indicate that both BC and MP possess substantial predictive ability in classifying between the classes. While BC is better at classifying the extremes (NC and AD), MP is more sensitive to progressive deterioration in abnormal cognitive aging, and hence, better for classifying EMCI and LMCI from controls. This attribute may allow MP (in contrast to BC), to be a better marker for identifying individuals with abnormal aging earlier in their trajectory.

## Discussion

Using RS-fMRI in subjects with progressive stages of MCI and AD with matched controls, we obtained directed brain networks using GC and estimated graph measures (BC and MP) from them. We hypothesized that these measures would progressively deteriorate from NC to EMCI to LMCI to AD. We found evidence for our hypothesis in L OFC (MP and BC) and LOC (MP) regions. Our primary findings were as follows. MP of two brain regions, LOC and L OFC, significantly decreased with the deterioration of the disease, while BC only decreased in LOC. In addition, no significant node was found in undirected networks (FC) using BC. MP of LOC and L OFC, and BC of LOC also exhibited significant associations with behavioral scores (MP had better association than BC), indicating their relevance to underlying pathology. Our results provide evidence that, for identifying imaging markers of deterioration from NC to MCI to DC, (i) MP is a better local nodal graph measure compared to BC and, (ii) MP/BC of directed networks seem to be more sensitive to disease progression than BC of undirected networks.

Our results are in agreement with previous functional network studies. The OFC is damaged conspicuously in AD, and from the view of neurofibrillary tangle (NFT) pathology, AD cases have pathology in OFC with distinct patterns of NFT while control cases have no appreciable pathology other than occasional NFT and diffused plaque [68]. OFC plays crucial roles in cognitive processing of decision-making [69] and age-related cognitive decline was shown to mirror neurodegenerative changes in this region [70]. On the other hand, LOC has also been previously noted in AD-related brain imaging studies. For example, it has been reported that with the deterioration of the disease, LOC showed a faster rate of atrophy in AD compared to MCI and NC [71]. Yao et al. found that FC between LOC and left amygdala decreased in EMCI compared to LMCI, and that the decrease in memory ability was related to such connectivity changes [72].

Next, we discuss the direction of network changes in MCI and AD (reduction in connectivity or graph measures with the progression of disease). In the introduction, we elaborated previous literature that has supported the dominant view of reduction in connectivity in MCI/AD, as well as pointed to a small number of studies that have also found increased connectivity. Since there has not been more direct and expansive evidence for the latter, we hypothesized that BC/MP of a few brain regions should progressively decrease with the deterioration of the disease. The deterioration hypothesis is a more mainstream view with wider acceptability, since it has roots in molecular/cellular level events in AD as discussed below.

Beta-amyloid (Aβ) shows a high degree of spatial overlap with default-mode network [73] and recent work has detected a linear relationship between amyloid deposition and FC derangement [74]. The Aβ is the critical initiating event in AD, starting with the aberrant clearance of Aβ-peptides followed by consecutive peptide aggregation and disruption of neural activity [75]. Thal et al. analyzed whole-brain regional Aβ deposition to assess differences in the expansion of Aβ-pathology between clinically proven AD cases and healthy population [20] and their results showed that occipital cortex and frontal cortex were severely affected by Aβ deposition with the deterioration of the disease. These results by Thal et al. are in concordance with our findings. Taken together, the reduction of MP/BC in MCI/AD is supported by deterioration in Aβ deposition with progression of disease. Given that estimating Aβ deposition requires a PET scan which is more invasive and expensive than an MRI scan, our results highlight the possibility of using the graph-theoretic characterization of directional brain networks obtained from RS-fMRI for tracking neurodegeneration.

Some other regions have also been reported to be crucial to AD pathology [11, 76, 77]. In fact, we also identified cingulate gyrus, hippocampus and middle temporal gyrus in 3 of the 6 comparisons (NC > EMCI, NC > AD, EMCI > AD) using MP. This is in accordance with previous studies [11, 76–78]. However, these regions were not identified in the remaining 3 comparisons involving LMCI (NC > LCMI, EMCI > LMCI, LMCI > AD). Considering that we were primarily interested in brain regions that progressively deteriorated from NC to EMCI to LMCI to AD, we did not emphasize these results in this report.

To clinically utilize our results, the findings must be replicated on a much larger sample, which is representative of the target population (gender, ethnicity, etc.). Future studies could choose to assess dynamic connectivity in addition to static connectivity as done in this study, which might provide further insights. Machine learning classifiers could be tested to develop MP/BC as biomarkers for prediction of disease progression at the single-subject level.

## Conclusion

In conclusion, our results showed that MP (estimated from GC networks) detected more brain regions that progressively deteriorated from NC to EMCI to LMCI to AD and had better association with behavioral variables, as compared to BC. Also, BC (estimated from FC networks) did not identify a single node, underscoring the superiority of GC over FC in our case. Our study provides evidence for the superiority of MP over BC and GC over FC. Estimated from GC networks, MP in L OFC and LOC could serve as potential biomarkers for progressive deterioration from NC to MCI to AD.

## Data Availability

The datasets used in this study were taken from the publicly available ADNI database, and are available for download from their website (http://www.loni.ucla.edu/ADNI).

## Abbreviations

Aβ: beta-amyloid
AD: Alzheimer’s disease
ADNI: Alzheimer’s disease neuroimaging initiative
BC: betweenness centrality
CDR: clinical dementia rating
CPGC: correlation-purged granger causality
CPL: characteristic path length
DPARSF: Data Processing Assistant for Resting-State fMRI
EMCI: early mild cognitive impairment
EPI: echo-planar imaging
FAQ: Functional Assessment Questionnaire
FC: functional connectivity
FMRI: functional magnetic resonance imaging
HRF: hemodynamic response function
LMCI: late mild cognitive impairment
LOC: lateral occipital cortex
MCI: mild cognitive impairment
MMSE: mini-mental state examination
MP: middleman power
MVAR: multivariate vector autoregressive model
NC: normal control
NFT: neurofibrillary tangle
NPI-Q: Neuropsychiatric Inventory Questionnaire
OFC: orbitofrontal cortex
ROI: region-of-interest
RS-fMRI: resting-state functional magnetic resonance imaging
SPM: statistical parametric mapping
